# Racial and Ethnic Disparities in Health Status and Community Functioning Among Persons with Untreated Mental Illness

**DOI:** 10.1007/s40615-022-01397-1

**Published:** 2022-09-06

**Authors:** Lonnie R. Snowden, Katharan Cordell, Juliet Bui

**Affiliations:** 1 School of Public Health, University of California, Berkeley, Berkeley, CA 94720 USA; 2 Center for Innovation and Population Health, University of Kentucky, Lexington, KY 40506 USA; 3 Office of Minority Health, U S Department of Health and Human Services, Rockville, MD USA

**Keywords:** Racial ethnic disparities, Community functioning, Health, Unemployment, Poverty, Criminal justice

## Abstract

Using  6 years of data from the National Survey of Drug Use and Health, the present study investigated ethnic minority-White disparities in self-rated health and community functioning for persons with untreated mental illness. Comparing minority and White persons with untreated severe mental illness (SMI) and mild and moderate mental illness (MMMI), the study sought evidence of “double jeopardy”: that minority persons with mental illness suffer an added burden from being members of ethnic minority groups. For African Americans with SMI and MMMI, results indicated that the odds were greater of living in poverty, being unemployed, and being arrested in the past year, and for African Americans with SMI, the odds were greater of reporting fair/poor health. For Native Americans/Alaska Native persons with MMMI, the odds were greater of living in poverty and being arrested in the past year. For Latinx persons with SMI and MMMI, the odds were greater of living in poverty and for Latinx persons with SMI the odds were greater of reporting fair/poor health. Results indicate that African Americans with mental illness suffer pervasive adversity relative to Whites and Native Americans/Alaska Natives and Latinx persons do so selectively.

 Ethnic minority persons with mental illness in the USA are less likely than Whites to receive treatment [1], and untreated mental illness brings challenges from mental illness’ functional impairment and stigma as well as from racial and ethnic prejudice and discrimination directed at minority persons. The greater health and social impact from minority status and mental illness is considered “double jeopardy”: when persons “who already confront prejudice and discrimination for their group affiliation, suffer double stigma when faced with the burdens of mental illness” [2].

Ethnic minority persons do confront racial and ethnic bias [3, 4] and mental illness is indeed stigmatizing [5] and disabling [6–8]. In fact, mental illness impairs health and creates difficulties in successful community functioning so much that mental illnesses rank among the leading contributors to the functioning-based global burden of disease [9]. However, that mental illness and minority-related challenges are significant barriers individually does not prove double jeopardy: the required compounding of the separate adversities need not occur [10–12].

## Proving Double Jeopardy: Methodological Challenges

Double jeopardy’s logic is appealing because “single jeopardy” from race and ethnicity and from mental illness clearly exists and because it is plausible that these challenges would combine for a joint impact greater than that for either alone. But, although it is not usually acknowledged [1, 2], researchers must define double jeopardy explicitly and collected evidence suited to document the implied joint effects.

Researchers also must clarify the methodological requirements for obtaining clear-cut evidence of double jeopardy. One approach is to identify samples of persons with mental illness—to control for mental illnesses as single jeopardy—and demonstrate added jeopardy from race and ethnicity by showing racial and ethnic disparities in health and functioning among persons with mental illness. Only one study of double jeopardy has been published thus far and it follows this approach. There, researchers demonstrated that, for persons with severe mental illness, African Americans and Latinx persons’ rates of diabetes were higher than Whites’ rates [13], thereby proving added risk of diabetes for ethnic minority persons beyond the risk alone from severe mental illness. Alternative approaches, including those controlling for underlying differences in minority and White persons who are not mentally ill, would further focus inquiry on *mental illness*-minority disparities.

## The Present Study: For Persons with Mental illness, Investigating Health Status, and Community Functioning Disparities Nationwide

The present study assessed double jeopardy—whether persons with mental illness suffer an added, ethnicity-associated jeopardy for less healthy and successful community functioning when they are members of largest ethnic minority groups. The investigators defined double jeopardy to occur when minority persons with mental illness suffer greater health and community functioning adversity than Whites going beyond disparities in health and community functioning in the general population.

The study examined this question in a large multiyear national probability sample of African Americans, Native Americans/Alaska Natives, Latinx, and Asian American/Pacific Islander/Native Hawaiian (AA/PI/AN), and Whites by asking whether, when predicting poverty, unemployment, self-rated fair or poor health, and being arrested in the past year, there are mental illness-specific racial and ethnic vs. White disparities in health and community functioning. The study’s comprehensive approach, featuring the largest ethnic groups and people with diagnosed mental illness, highlights key areas of functioning where mental illness has been associated with considerable adversity: higher poverty [14] and unemployment rates [15], poor health and a shorter life expectancy [6], and criminal justice involvement [7].

 Two levels of mental illness were considered: severe mental illness (SMI) and mild and moderate mental illness (MMMI). This differentiation is important because some theories imply that symptoms of SMI—hallucinations, for example—are so prominent and disruptive that they preempt anti-minority prejudice and discrimination [10, 16–18]. The present study examines whether disparities are greater for persons with MMMI than with SMI.

Several steps were taken to isolate untreated SMI’s and MMMI’s impact on disparities in health status and community functioning. To eliminate the influence of White’s higher mental illness treatment rates [20], the study selected persons with untreated mental illness. The study controlled for co-occurring substance abuse and for key population demographic differences and, as previously discussed, controlled for disparities in health and community functioning in the population of persons without diagnosed mental illness.

## Methods

Utilizing Substance Abuse and Mental Health Services Administration’s National Survey of Drug Use and Health’s NSDUH, the present study estimated the odds of AI/AN, African Americans’, Latinos’, versus Whites’ detrimental health, economic, and criminal justice experiences nationwide and over several years, while adjusting for population differences in substance abuse, age, gender, and residence (urban vs. rural), and for survey sampling factors. National population estimates assess the magnitude of disparities in the USA for racial/ethnic minorities and Whites with untreated SMI and MMMI beyond disparities among persons without mental illness.

### The National Survey of Drug Use and Health

The National Survey of Drug Use and Health (NSDUH) is a nationally representative, annual survey of approximately 67,500 individuals. The NSDUH does not sample persons who are non-sheltered homeless, active-duty military, or institutionalized in jails or hospitals. The present sample aggregated six cross-sectional survey years (2009–2014) including approximately 204,000 respondents ages 18–64 with complete data. The full sample used in preliminary analysis included approximately 9800 AANHPI, 3300 AI/AN, 26,800 African American, 34,900 Latino, and 129,600 White respondents. Persons meeting criteria for SMI and MMMI used in final analysis included approximately 21,100 individuals with untreated MMMI, and 3700 with untreated SMI.

#### SMI and MMMI

The NSDUH assesses “any mental illness,” defined as having a diagnosable mental, behavioral, or emotional disorder other than a developmental or substance use disorder as assessed by the Mental Health Surveillance Study Structured Clinical Interview for the Diagnostic and Statistical Manual of Mental Disorders—Fourth Edition—Research Version—Axis I Disorders, based on the 4th edition of the Diagnostic and Statistical Manual of Mental Disorders (DSM-IV). Responses are solicited on the Kessler-6 scale of psychological distress [21], and the World Health Organization Disability Assessment Schedule for assessing functional impairment [22], and any mental illness (AMI) is scored from statistical models predicting the presence of AMI in survey subsamples completing a Structured Clinical Interview for DSM-IV Axis I Disorders [23].

SMI is having a mental illness as defined above resulting in serious functional impairment. Assignment to the SMI category is based not on direct measures of diagnostic status but on a predictive model. MMMI consists of persons determined to have any mental illness but not SMI.

#### Living in Poverty

The NSDUH determines poverty status by soliciting family income and subsequently classifying respondents based on number of children and other factors according to national poverty thresholds published by the U.S. Census Bureau. Respondents were classified as living in poverty (family income less than 100% of the poverty threshold) or not living in poverty.

#### Unemployed

Respondents were identified as unemployed if they were not employed in the last week and were looking for a job. Respondents who were employed full time, employed part time, or not seeking employment were identified as not unemployed.

#### Poor/Fair Health Status

Health status was assessed by “This question is about your overall health. Would you say your health in general is excellent, very good, good, fair, or poor?” Respondents who answered fair or poor were classified as such. Respondents not in poor/fair health answered as excellent, very good, or good.

#### Past-Year Arrest

Survey respondents were identified as having a past-year arrest if they reported having been arrested one or more times in the last 12 months.

#### Race and Ethnicity

Survey respondents who selected Hispanic as their ethnicity were categorized as Latinx. Non-Hispanic White and Black persons were categorized as White and African American, respectively. Persons selecting American Indian or Alaska Native were classified as such. Persons selecting Non-Hispanic Native Hawaiian, Pacific Islander, Chinese, Filipino, Japanese, Asian Indian, Korean, Vietnamese, Other Asian, and Multiple Asian categories were classified as Asian American/Native Hawaiian/Pacific Islander.

Respondents selecting more than one race or ethnicity were classified by the NSDUH as “other.” To focus on people identified most closely with each ethnic group and to forestall justifiable but, for present purposes, overly complex interpretations of ethnicity, we omitted persons whose race or ethnicity was classified as “other” from our sample.

#### Demographic Controls

We adjusted for differences in population structure. Gender was identified as male or female. Age groups were 18–25, 26–34, 35–49, and 50–64. Respondent’s counties of residence, if non-metropolitan, were classified as rural. Respondents living in small or large metropolitan were identified as urban. The year that the respondent was surveyed, 2009 through 2014, also served as a control.

### Analysis

To assess living in poverty, unemployed, poor/fair health status, and being arrested in the past year and comparing each racial/ethnic minority group with Whites, the investigators created proportional estimates accounting for the survey’s complex sampling design. Condition rates were calculated using SAS Survey means procedure while considering the weighting, clustering, and stratification of the design within each race/ethnic group [24]. The resulting rates consider the representation of survey respondents relative to corresponding national population representations and estimate the rate of each condition in each racial/ethnic group. Population proportions were estimated by race/ethnicity for all individuals nationally as well as for individuals with untreated mental illness versus those without an identified mental illness or who were treated. All analyses were performed using Statistical Analysis Software (SAS) 9.3.

#### Odds Ratio Computation

Health and community functioning indicators were regressed on mental illness and race/ethnicity while controlling for gender, age group, rural county of residence, and survey disparities year. Racial and ethnic vs. White disparities were identified as significant interactions between race/ethnicity vs. White status and untreated MMMI or SMI. These interactions demonstrated “double jeopardy”: that MMMI and SMI disparities exceeded those found among persons without a mental disorder or undergoing treatment.

The investigators estimated a total of four weighted logistic regression models for SMI and four for MMPI to produce adjusted odds ratios. Odds compared living in poverty, for example, for ethnic/racial minorities as compared to White when afflicted with an untreated mental illness, equalizing on control variables (full results not shown). Weighted logistic regression models were developed using SurveyLogistic in Statistical Analysis Software (SAS) 9.3,

#### Population Proportions

Population proportion estimates were created for each racial/ethnic minority group while accounting for the survey’s complex sampling design. The proportion of the nationwide population experiencing each outcome was calculated using SAS SurveyMeans procedure while considering the weighting, clustering, and stratification of the survey design within the domains of race/ethnicity [24]. The resulting proportions consider the representation of survey respondents relative to the national population and estimate the proportion of individuals within each racial/ethnic group living in the USA who experience each concern. Results of population proportions are displayed alongside logistic regression results in Tables 2 and 3.

## Results

Descriptive statistics are presented in Table 1. Whites made up about 63.5% (*n* = 114,167) of the sample, African Americans about 13.1% (*n* = 23,507), Latinx 17.1% (*n* = 30,689), Asian American/Native Hawaiian/Pacific Islanders about 4.7% (*n* = 8483), and American Indian/Alaska Natives about 1.6% (*n* = 2818).

**Table 1:** NSDUH sample description

	Treated/no MI		MMMI-untreated		SMI-untreated		Total
	*N*	Sample %	*N*	Sample %	*N*	Sample %	*N*
All	179,664	100.0	21,095	100.0	3654	100.0	204,413
Gender							
Male	85,236	47.4	8673	41.1	1446	39.6	95,355
Female	94,428	52.6	12,422	58.9	2208	60.4	109,058
Race/ethnicity							
White	114,167	63.5	13,140	62.3	2339	64.0	129,646
African American	23,507	13.1	2819	13.4	460	12.6	26,786
Latinx	30,689	17.1	3582	17.0	622	17.0	34,893
AANHPI	8483	4.7	1178	5.6	155	4.2	9816
AI/AN	2818	1.6	376	1.8	78	2.1	3272
Age group							
18–25 years old	85,091	47.4	10,492	49.7	1,880	51.5	97,463
26–34 years old	30,171	16.8	4165	19.7	727	19.9	35,063
35–49 years old	42,833	23.8	4615	21.9	789	21.6	48,237
50–64 years old	21,569	12.0	1823	8.6	258	7.1	23,650
County type							
Non-rural county	142,576	79.4	16,842	79.8	2886	79.0	162,304
Rural county	37,088	20.6	4253	20.2	768	21.0	42,109
Year of survey							
2009	29,042	16.2	3611	17.1	588	16.1	33,241
2010	30,307	16.9	3570	16.9	586	16.0	34,463
2011	30,323	16.9	3524	16.7	619	16.9	34,466
2012	29,255	16.3	3460	16.4	614	16.8	33,329
2013	28,930	16.1	3385	16.1	616	16.9	32,931
2014	31,807	17.7	3545	16.8	631	17.3	35,983
Poverty level							
Living in poverty	37,171	20.7	5120	24.3	1048	28.7	43,339
Up to 2 × Fed Pov Thresh	40,149	22.4	5141	24.4	1022	28.0	46,312
> 2 × Fed Pov Thresh	102,344	57.0	10,834	51.4	1584	43.4	114,762
Past week unemployment							
Not unemployed	163,704	91.1	18,824	89.2	3130	85.7	185,658
Unemployed	15,960	8.9	2271	10.8	524	14.3	18,755
Health status							
Good or better health	164,402	91.5	18,309	86.8	2880	78.8	185,591
Poor or fair health	15,262	8.5	2786	13.2	774	21.2	18,822
Past year crime							
No	171,704	95.6	19,837	94.0	3343	91.5	194,884
Yes	7960	4.4	1258	6.0	311	8.5	9529

 Regression results indicating racial and ethnic disparity by SMI and MMMI interactions are presented in Tables 2 and 3. Results for poverty and unemployment are presented in Table 2 and for fair/poor health and past year arrest in Table 3.

**Table 2:** Poverty and unemployment: odds and population proportions for persons with untreated mental illness by race/ethnicity

2009–2014		Living in poverty				Unemployed				
		Odds ratio*	95% CI	Pop. prop	95% CI		Odds ratio*	95% CI	Pop. prop	95% CI
Mental illness status										
Untreated SMI		**2.3**	**(2.0–2.6)**	**25%**	**(23–27)**		**2.1**	**(1.8–2.5)**	**11%**	**(10–12)**
Untreated MMMI		**1.5**	**(1.4–1.6)**	**20%**	**(19–20)**		**1.3**	**(1.2–1.4)**	**8%**	**(8–9)**
Treated or no MI (ref.)		1.0		15%	(14–15)		1.0		6%	(6–7)
Race/ethnicity										
AANHPI		**1.4**	**(1.2–1.5)**	**13%**	**(12–14)**		1.0	(0.8–1.1)	5%	(5–6)
AI/AN		**4.0**	**(3.3–4.8)**	**35%**	**(31–38)**		**2.2**	**(1.9–2.7)**	**12%**	**(10–14)**
African American		**3.5**	**(3.3–3.7)**	**28%**	**(27–29)**		**2.2**	**(2.1–2.4)**	**12%**	**(11–12)**
Latinx		**3.3**	**(3.1–3.5)**	**26%**	**(25–27)**		**1.4**	**(1.3–1.5)**	**8%**	**(7–8)**
White (ref.)		1.0		10%	(10–11)		1.0		5%	(5–6)
Mental illness status and race/ethnicity interaction										
Untreated SMI by race/ethnicity										
AANHPI		1.0	(0.5–1.9)	20%	(11–29)		0.7	(0.2–2.2)	8%	(1–15)
AI/AN		2.1	(0.9–5.1)	38%	(22–55)		1.3	(0.4–4.0)	15%	(3–27)
African American		**2.8**	**(1.9–3.9)**	**41%**	**(35–47)**		**1.6**	**(1.0–2.5)**	**16%**	**(12–21)**
Latinx		**1.7**	**(1.1–2.5)**	**31%**	**(25–37)**		0.8	(0.4–1.3)	9%	(6–12)
White (ref.)		1.0		21%	(19–23)		1.0		11%	(9–13)
Untreated MMMI by race/ethnicity										
AANHPI		1.1	(0.7–1.7)	16%	(12–19)		1.1	(0.6–2.0)	8%	(5–10)
AI/AN		**3.6**	**(1.3–7.4)**	**40%**	**(30–50)**		2.0	(0.8–4.3)	13%	(8–19)
African American		**3.3**	**(1.9–4.1)**	**35%**	**(31–38)**		**2.5**	**(1.7–3.2)**	**15%**	**(13–17)**
Latinx		**2.3**	**(1.4–2.9)**	**28%**	**(25–31)**		1.3	(0.9–1.8)	9%	(8–11)
White (ref.)		1.0		14%	(13–15)		1.0		7%	(6–7)

^*^Adjusting for gender, age group, rural county, year of survey; Bolded: *p *>.01

**Table 3:** Fair/poor health and arrest in the past year: odds and population proportions for persons with untreated mental illness by race/ethnicity

2009–2014		Poor/fair health				Arrested in past year							
		Odds ratio*	95% CI	Pop. prop	95% CI	Odds ratio*		95% CI		Pop. prop		95% CI	
Mental illness													
Untreated SMI		**4.4**	**(3.8–5.1)**	**29%**	**(26–31)**	**3.0**		**(2.3–3.9)**		**8%**		**(6–9)**	
Untreated MMMI		**2.1**	**(1.9–2.3)**	**17%**	**(16–18)**	**1.4**		**(1.2–1.6)**		**4%**		**(4–5)**	
Treated or no MI (ref.)		1.0		11%	(10–11)	1.0				3%		(3–3)	
Race/ethnicity													
AANHPI		0.8	(0.7–1.0)	7%	(6–8)	0.3		(0.2–0.3)		< 1%		(< 1– < 1)	
AI/AN		**2.7**	**(2.2–3.3)**	**22%**	**(19–26)**	**3.9**		**(3.2–4.9)**		**10%**		**(8–12)**	
African American		**1.8**	**(1.7–2.0)**	**15%**	**(14–15)**	**2.1**		**(1.9–2.4)**		**5%**		**(5–6)**	
Latinx		**2.4**	**(2.2–2.5)**	**16%**	**(15–17)**	1.1		(1.0–1.3)		3%		(3–4)	
White (ref.)		1.0		10%	(10–10)	1.0				3%		(2–3)	
Mental illness status and race/ethnicity interaction													
Untreated SMI by race/ethnicity													
AANHPI		1.0	(0.4–2.4)	23%	(10–36)	0.1			(0.0–0.3)		< 1%		(0– < 1)
AI/AN		1.3	(0.4–3.6)	30%	(10–50)	0.9			(0.2–3.0)		7%		(0–14)
African American		**1.6**	**(1.0–2.4)**	**32%**	**(26–39)**	**2.1**			**(1.2–3.7)**		**13%**		**(8–17)**
Latinx		**1.9**	**(1.2–2.8)**	**35%**	**(28–41)**	1.0			(0.6–1.9)		8%		(4–11)
White (ref.)		1.0		27%	(24–29)	1.0					7%		(6–9)
Untreated MMMI by race/ethnicity													
AANHPI		0.7	(0.4–1.4)	10%	(6–14)		0.2		(0.1–0.4)		1%		(0–1)
AI/AN		1.8	(0.5–3.8)	26%	(16–36)		**4.5**		**(1.4–11.7)**		**15%**		**(5–24)**
African American		1.6	(0.6–2.1)	21%	(18–24)		**2.4**		**(1.4–3.4)**		**7%**		**(6–9)**
Latinx		1.9	(0.8–2.5)	22%	(19–24)		1.3		(0.7–2.0)		5%		(4–6)
White (ref.)		1.0		16%	(15–17)		1.0				3%		(3–4)

^*^Adjusting for gender, age group, rural county, year of survey; Bolded: *p *>.01

Most odds ratios favored Whites over minorities, and several were statistically significant. African Americans with SMI’s odds of living in poverty were 2.8 times greater than White’s odds (95% CI = 1.9, 3.9); their odds of being unemployed were 1.6 times greater (95% CI = 1.0, 2.5), their odds of self-rated fair/poor health were 1.6 times greater (95% CI = 1.0, 2.4), and their odds of arrest in the past year were 2.1 times greater (95% CI = 1.2, 3.7). Latinx odds of living in poverty were 1.7 times greater than Whites’ odds (95% CI = 1.1, 2.5) and their odds of self-rated air/poor health were 1.9 times greater (95% CI = 1.2, 2.8).

African Americans with MMMI’s odds of living in poverty were 3.3 times greater than Whites’ odds (95% CI = 1.9, 4.1); their odds of being unemployed were 2.5 times greater (95% CI = 1.7, 3.2), and their odds of arrest in the past year were 2.4 times greater (95% CI = 1.4, 3.4). American Indian/Alaska Natives with MMMI’s odds of living in poverty were 3.6 times greater than Whites’ odds (95% CI = 1.3, 7.4), and odds of arrest in the past year were 4.5 times greater (95% CI = 1.4, 11.7). Latinx odds of living in poverty were 2.3 times greater than Whites’ odds (95% CI = 1.4, 2.9).

## Discussion

For African Americans with mental illness especially, prospects were significantly worse than Whites’ prospects for healthy and successful community living. African Americans with SMI and MMMI were more likely to be poor, unemployed, and arrested in the past year. Furthermore, African Americans with MMMI were more likely than Whites to report being in fair/poor health. Vulnerabilities from mental illness were amplified by African Americans’ other vulnerabilities stemming from more limited educational opportunities and greater residence in segregated neighborhoods of concentrated poverty, biased policing and excess incarceration, exposure to violence and victimization, lack of opportunities practice healthy eating and exercise habits, and lack of access to health insurance and other structural racism indicators. When combined with mental illness, these and other economic and social disadvantages produced even greater economic, health, and criminal justice adversity.

Native American/Alaska Native persons with MMMI, but not those with SMI, demonstrated considerably higher odds of living in poverty and of arrest: odds of impoverishment were more than three times greater than Whites’ odds and Native American/Alaska Natives’ odds of arrest more than four times greater. In absent prominent symptoms and disability from SMI, Native American/Alaska Natives people with MMMI were challenged considerably by mental illness more than Whites. Native American/Alaska Natives’ greater social and economic vulnerability and greater exposure to bias and discrimination became formidable obstacles when mental illness’ disability was less; with less disabling mental illness came greater challenges to earning capacity and more scrutiny and intervention by law enforcement officials. Mild and moderate mental illness intensifies these adversities especially for Native American/Alaska Native people.

Neither for SMI nor MMMI did Asian American/Pacific Islander/Native Hawaiian people show greater adversity than Whites in poverty, unemployment, fair/poor health, or arrest. Individual Asian American/Pacific Islander/Native Hawaiian subgroups vary widely in economic and social standing, and some are markedly disadvantaged; all face prejudice and discrimination. More personal and family financing resources, on average, may provide a better cushion against the challenges of mental illness. Research is needed to understand personal and community coping strategies permitting Asian American/Pacific Islander/Native Hawaiian people with mental illness not to economic and social adversity at levels characterizing other ethnic minority groups.

SMI and MMMI were associated with poverty disparities for Latinx persons and with fair/poor health for Latinx persons with SMI. Latinx persons’ labor market disadvantages and economically marginal position may cause them more readily to slip into poverty when mental illness interferes with their ability to meet customary job-related responsibilities. Lack of access to publicly supported programs for undocumented persons and, whether they are documented or not, concern about mistreatment by immigration officials may limit a willingness to express health-related concerns or exposure to police or other authority figures who might challenge Latinx people over their immigration status.

The present findings argue for a differentiated approach when formulating questions to address mental illnesses and ethnic minority status’s impact on adversity. While marginalization from race and ethnicity and mental illness is a legitimate source of universal concern, whether their impact is additive varies with the group, mental illness severity, and outcome under consideration. Theorists and researchers should focus on identifying specific pathway of influence by which ethnic identities and community experiences of minority-based and stigma introduce barriers to community well-being and successful living. Conceivably, disparities in individual and family living conditions and resources, labor market positioning, family and network resources, and local community characteristics—as well as immigration status, English language proficiency, and community and institutional stigma—all can be influential. Sweeping characterizations should give way to group-specific accounts applied to less and more severe forms of mental illness.

### Improving Behavioral and Physical Health and Promoting Community Adjustment

 More effort is warranted to address past deficiencies that have left too many minority persons untreated and otherwise prevented disproportionately many minority persons with mental illness from reaching their potential for healthy and productive living. Outreach to minority communities must be increased to identify persons with mental illness and link them with culturally informed treatment resources. Culturally attuned media campaigns targeting minority communities should be implemented, and community opinion leaders enlisted who can reach persons with mental illness and their families. Greater use should be made of community health workers [25] by health plans and community clinics for reaching out to reduce mental illness stigma and to identify persons with mental illness, referring them for appropriate treatment and navigating complex treatment bureaucracies. Social services bureaucracies are equally complex and, serving as navigators, community health workers can facilitate successful community functioning by bringing to minority persons with mental illness income and employment assistance and other sources of needed support.

Not-for-profit community-based organizations—non-governmental, civil society, or other grassroots organizations with a community service mission—can also play meaningful roles in reducing barriers to mental health treatment by reaching out to minority communities and reducing mental health treatment disparities. Ethnic minority people are overrepresented in Federally Qualified Health Centers (FQHCs) [26], which deliver a large and growing volume of mental health care. FQHCs are well-positioned to screen minority patients for mental health problems and undertake wide-scale stigma reduction efforts. Thousands of nonprofit hospitals have also become community hubs in return for state, federal, and local tax relief [27]. All not-for-profit health plans and hospitals should assess mental health needs in ethnic minority communities in their mandated community health needs assessments [28].

Ethnic minority persons with mental illness may be doubly discriminated against, and Federal, state, and local antidiscrimination policies should be examined to address this possibility. Fair employment and fair housing policies must recognize discrimination against minority people with mental illness. Policies requiring language assistance for persons with Limited English Proficiency in mental health settings—the Americans with Disabilities Act—may be a major leverage point for preventing discrimination against people with mental illness. Especially for persons with SMI who are disproportionately homeless and ethnic minority persons, Housing First policies improve residential stability [29]. Housing First can be indispensable, providing a foundation for achieving better health and successful community functioning. Vigilance is needed to insure that people with SMI are not discriminated against due to either SMI [30] or ethnic minority status.

### Limitations

Several limitations deserve mention. The NSDUH does not collect surveys from individuals who are non-sheltered homeless, active-duty military, or institutionalized in jails or hospitals. African and Native Americans and Latinos are overrepresented in several of these categories and their omission might bias the findings proportionately. Critics have challenged the cross-cultural validity of diagnostic assessment procedures, and sometimes of diagnosis itself [31, 32] introducing another possible source of bias.

Another limitation arises from the reciprocal influences between mental illness on the one hand and poverty, unemployment, health, and criminal justice involvement on the health and criminal justice involvement cause more minority’s than Whites’ mental illness rather than minorities’ and White’s mental illness causing more minority persons to suffer poverty, unemployment, poor health, and criminal justice involvement.

Despite these limitations, the study provides clear-cut evidence that for some groups and some disorders, race and ethnicity are associated with disparities in health, economic, and criminal justice involvement. Double jeopardy is selective, but it does exist, doubly disadvantaging some minority persons with mental illness. Only from a wide-ranging and vigorous effort can the special burden of impaired health and community functioning be reduced for ethnic minority persons.
